# Velamentous Cord Insertion in a Singleton Pregnancy: An Obscure Cause of Emergency Cesarean—A Case Report

**DOI:** 10.1155/2012/308206

**Published:** 2012-11-29

**Authors:** Juliana Rocha, Joana Carvalho, Fernanda Costa, Isabel Meireles, Olímpia do Carmo

**Affiliations:** Department of Gynecology and Obstretics, Tâmega e Sousa Hospital, Lugar do Tapadinho, Guilhufe, 4564-007 Penafiel, Portugal

## Abstract

Approximately 2% of low-risk pregnant women still require an emergency Cesarean section after the onset of labor. Because it is likely that half of these cases are associated with placental and umbilical cord abnormalities, it is thought that prenatal detection of such abnormalities would reduce the number of emergency cesarean sections in low-risk women. Velamentous cord insertion is an abnormal cord insertion in which the umbilical vessels diverge as they traverse between the amnion and chorion before reaching the placenta. With a reported incidence of 1% in singleton pregnancies, it has been associated with several obstetric complications. This condition has been diagnosed by ultrasonography with a sensitivity of 67% and specificity of 100% in the second trimester. The present case highlights the importance of the systematic assessment of the placental cord insertion site at routine obstetric ultrasound and the potential of identifying pregnancies with velamentous insertion and, therefore, those at risk for obstetric complications.

## 1. Introduction

The umbilical cord normally inserts into the central portion of the placenta, well away from the placental edge. The site of placental cord insertion (CI) is considered abnormal when it is located at the edge of the placental disk or when the umbilical vessels separate from each other and course between the amnion and chorion before reaching the placenta (velamentous CI, VCI) [1].

Perinatal deaths are most frequent in pregnant women with abnormalities of the placenta, umbilical cord, and fetal membrane. Despite advances in perinatal medicine, approximately 2% of low-risk pregnant women still require an emergency cesarean section after the onset of labor. Because it is likely that half of these cases are associated with placental and umbilical cord abnormalities, it is thought that prenatal detection of such abnormalities would reduce the number of emergency cesarean sections in low-risk women [1].

Velamentous cord insertion (VCI) is an abnormal cord insertion (CI) in which the umbilical vessels diverge as they traverse between the amnion and chorion before reaching the placenta. It is characterized by membranous umbilical vessels at the placental insertion site; the remainder of the cord is usually normal. Because of the lack of protection from Wharton's jelly, these vessels are prone to compression and rupture, especially when they are located in the membranes covering the cervical ostium (vasa previa). The length of the membranous vessels or the distance between the end of the normal cord and the placental insertion is highly variable [2].

Pregnancies complicated with VCI are at greater risk for adverse perinatal outcome (fetal growth restriction, preterm labor, placental abruption, vasa previa, abnormal intrapartum fetal heart rate (FHR) patterns, low Apgar scores at 1 and 5 minutes, and neonatal death) [3, 4]. During intrapartum, variable decelerations and a non-reassuring FHR pattern are frequently observed in cases with VCI [1]. 

Investigators have suggested that the systematic identification of the abnormal CI is an extremely important part of prenatal ultrasound evaluation [5–7]. 

This condition has been diagnosed by ultrasonography with a sensitivity of 67% and specificity of 100% in the second trimester [1]. 

On ultrasound and gross examination, the normal umbilical cord sheath is contiguous with the chorionic plate. With a velamentous insertion, the cord can end several centimeters from the placenta, at which point the umbilical vessels separate from each other and cross between the amnion and chorion before connecting to the subchorionic vessels of the placenta. Color Doppler imaging enhances identification of the vessels [2]. 

Prenatal detection of VCI reduces the number of emergency Cesarean sections in low-risk women, and thus may also reduce fetal and neonatal morbidity and mortality [1]. 

The present case is valuable to reflect the importance of prenatal diagnosis of this clinical scenario. 

## 2. Case Report

A 35 year old woman, gravid 2, para 1, with a 41 weeks low-risk gestation during the screening scan, was admitted to our hospital for labor induction with intravaginal dinoprostone. Of note, that transabdominal ultrasonography at 21 weeks' gestation, the placenta was located on the posterior wall with no abnormalities detected. There was also no reference of an abnormal fetal growth. 

Fetal heart rate was monitored continuously (department's protocol). Vaginal examination at admission indicates a posterior cervix, 2 cm of dilatation with no effacement. No significant anomalies were detected during monitoring. Spontaneous membrane rupture took place after 9 hours. 

**Figure 1:**
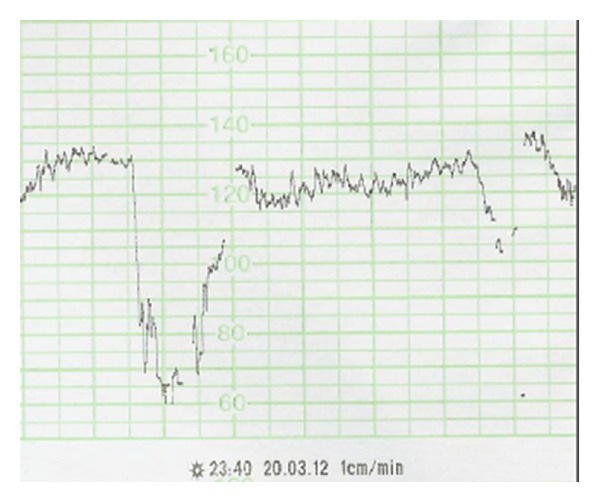
Fetal heart rate pattern showing variable deceleration with no acceleration (VDna).

At 14 hours after induction, cardiotocography reveals atypical variable decelerations (example in Figure 1). 

**Figure 2:**
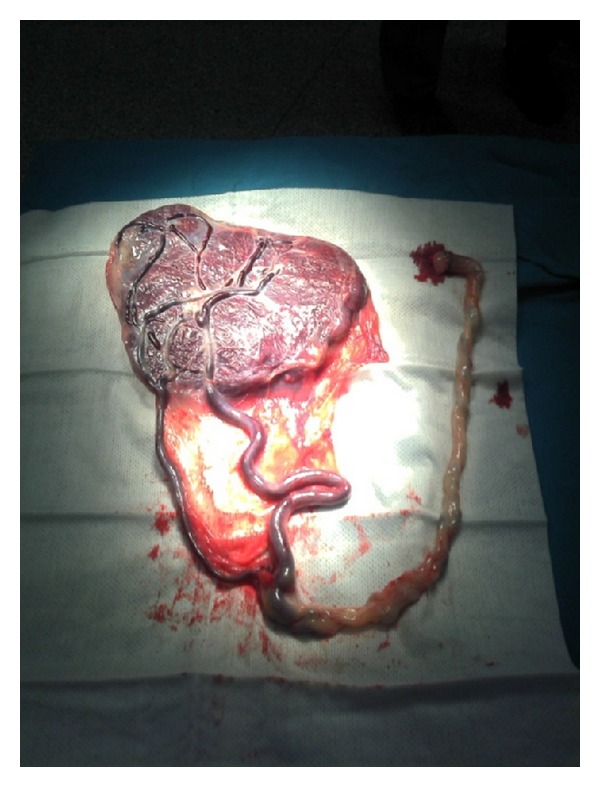
Photograph showing velamentous cord insertion of the placenta.

Considering that the patient was remote from delivery (6 cm cervical dilatation); a decision for emergency cesarean delivery was taken. The intervention took place without registration of problems. During surgery, a posterior discoid placenta with velamentous cord insertion and around 15 cm of vessels traversing in membranes were observed (Figure 2).

A female infant was delivered, weighing 2542 gr, with Apgar scores of 8 and 9 at 1 and 5 min. There was no record of neonatal or obstetric complications.

## 3. Discussion

Velamentous insertion occurs in approximately 1 percent of singleton gestations [5], but is observed in as many as 15 percent of monochorionic twin gestations [8–10]. 

It is also more common in placenta previa than in normally located placentas. The pathogenesis is still unknown [11, 12]. In small series and case reports, velamentous insertion has been described in association with several obstetrical complications [1, 3, 4, 10–15]. It is thought that frequent FHR abnormalities in VCI cases are caused by a lack of Wharton's jelly, which results in compression of VCI vessels during uterine contractions [3, 4, 16, 17]. 

Cord occlusion, either partial or complete, can cause both increases in afterload and decreases in fetal arterial oxygen content, both of which will result in an activated vagal reflex causing bradycardia [18]. 

Atypical variable decelerations (VD) was reported by Krebs et al. [19] in 1983 as prognostically unfavorable with features indicative of fetal hypoxia, including the slow return of the FHR to the baseline, loss of variability during the deceleration, loss of initial and/or secondary accelerations, persistence of secondary acceleration (overshoot), continuation of the FHR at a lower level, and biphasic deceleration. 

Lee et al. [20] suggested that pure VDs are typically caused by different degrees of partial cord compression. Usually, when compression of the umbilical cord occurs, the umbilical vein is obstructed first, reducing fetal blood return and inducing acceleration of the FHR via baroreceptors. Subsequent complete obstruction of both umbilical arteries and veins induces fetal systemic hypertension, which results in the deceleration of the FHR. After deceleration, when the compression is released, acceleration is observed as the order of events is reversed. Variable deceleration without baroreceptor-mediated acceleration (VDna) frequently occurs in VCI cases. The cause of frequent and early appearance of VDna is thought to be the compression of aberrant vessels, which are not coated or thinly coated with Wharton's jelly, and the blood flow of both umbilical arteries and veins would be obstructed at the same time during uterine contractions or fetal movement [3, 16, 21]. Analysis of VDna occurrence in the second stage of labor shows that the rate of VDna is about three times higher in VCI cases than in controls [21]. 

Clark [17] reported that a significant aspect of FHR monitoring in VCI cases was marked decelerations with membranes intact without vaginal bleeding.

The prenatal diagnosis of velamentous insertion is based upon the presence of characteristic sonographic findings (membranous umbilical vessels) at the placental cord insertion site, using gray-scale ultrasound [22], color Doppler [5], and three-dimensional ultrasound [23]. The criteria for ultrasound diagnosis of a VCI are: umbilical vessels entering the placenta margin parallel to the uterine wall and connecting to superficial placental vessels; an immobile CI, even when the uterus is shaken; and umbilical vessels diverging as they traverse the membrane. In fact, the CI site could not be determined more frequently in cases of marginal CI and VCI than in the normal CI [1]. When color Doppler is used to enhance identification of the vessels, diagnostic sensitivities of 69 to 100 percent and specificities of 95 to 100 percent have been reported [5, 24]. 

A definitive diagnosis is made by gross examination of the placenta, cord, and membranes after delivery.

A recent report indicated that 85% of obstetricians in England and Wales stated that VCI is not routinely screened during anomaly scanning [25]. It is believed that possible reasons for the lack of VCI detection during screening may be difficulty in recognizing the condition and the time required for evaluation of the cord insertion.

Even when this is done, the diagnosis of velamentous insertion may not be made in the prenatal period, and failure to make this diagnosis is not a gap of the standard of care [14]. 

In VCI, the umbilical arteries and vein branch run into several large vessels soon after the cord insertion into the fetal membranes, and these vessels run between the insertion site and the placenta. This forms a specific feature of this condition, and thus not only the insertion site itself but also the branching vessels running within the fetal membranes should be targeted in the sonographic identification of VCI [26]. 

Pretorius et al. [27] reported that the detection rate of CI was markedly influenced by gestational age, ranging from 67% at 15–20 weeks to 30% at 36–40 weeks. As visualization of the placental CI site becomes more difficult with advancing gestation, it should be evaluated in the second trimester [5, 6, 28]. The sensitivity of diagnosing a VCI was also low when the CI was located on the posterior wall. However, using routine color Doppler scanning, Sepulveda et al. [5] and Nomiyama et al. [6] identified the placental cord insertion site in more than 99% of their cases. In all cases of less than 30 weeks' gestation, they were able to identify the CI regardless of placenta location [5]. In their studies, ultrasound scans were performed by a single fetal medicine specialist.

An abnormal CI was present more frequently when it was difficult to image the CI (decreased sensitivity); thus, a more precise scan (scanning in different body positions and using color Doppler), as utilized in other studies, is indicated [5, 27]. 

There are no data from large or controlled studies on which to base management recommendations. 

## 4. Conclusion

Through our management of pregnant women, it is believed that a safer delivery can be provided with accurate identification of high-risk pregnancies with abnormalities of the placenta and umbilical cord. 

Since VCI was not identified prenatally and many of its sequelae are readily identifiable only during the intrapartum period, the potential for preemptive obstetric intervention appears to be limited. 

Abnormal cord insertions are associated with increased rates of abnormal FHR tracings and Cesarean deliveries. In particular, a VCI should be deemed a high-risk pregnancy and a warning sign of a possible vasa previa. Despite the favorable outcome of this case, it allows to highlight the importance of antenatal diagnosis of a VCI in early gestation and possibly improve obstetric management.
